# Aberrant Glycogen Synthase Kinase 3β Is Involved in Pancreatic Cancer Cell Invasion and Resistance to Therapy

**DOI:** 10.1371/journal.pone.0055289

**Published:** 2013-02-08

**Authors:** Ayako Kitano, Takeo Shimasaki, Yuri Chikano, Mitsutoshi Nakada, Mayumi Hirose, Tomomi Higashi, Yasuhito Ishigaki, Yoshio Endo, Takahisa Takino, Hiroshi Sato, Yoshimichi Sai, Ken-ichi Miyamoto, Yoshiharu Motoo, Kazuyuki Kawakami, Toshinari Minamoto

**Affiliations:** 1 Division of Translational and Clinical Oncology, Cancer Research Institute, Kanazawa University, Kanazawa, Japan; 2 Division of Molecular Virology and Oncology, Cancer Research Institute, Kanazawa University, Kanazawa, Japan; 3 Central Laboratory, Cancer Research Institute, Kanazawa University, Kanazawa, Japan; 4 Department of Hospital Pharmacy, Graduate School of Medical Science, Kanazawa University, Kanazawa, Japan; 5 Department of Neurosurgery, Graduate School of Medical Science, Kanazawa University, Kanazawa, Japan; 6 Department of Hygiene, Graduate School of Medical Science, Kanazawa University, Kanazawa, Japan; 7 Department of Medical Oncology, Kanazawa Medical University, Uchinada, Ishikawa, Japan; 8 Medical Research Institute, Kanazawa Medical University, Uchinada, Ishikawa, Japan; University of Kansas Medical Center, United States of America

## Abstract

**Background and Purpose:**

The major obstacles to treatment of pancreatic cancer are the highly invasive capacity and resistance to chemo- and radiotherapy. Glycogen synthase kinase 3β (GSK3β) regulates multiple cellular pathways and is implicated in various diseases including cancer. Here we investigate a pathological role for GSK3β in the invasive and treatment resistant phenotype of pancreatic cancer.

**Methods:**

Pancreatic cancer cells were examined for GSK3β expression, phosphorylation and activity using Western blotting and *in vitro* kinase assay. The effects of GSK3β inhibition on cancer cell survival, proliferation, invasive ability and susceptibility to gemcitabine and radiation were examined following treatment with a pharmacological inhibitor or by RNA interference. Effects of GSK3β inhibition on cancer cell xenografts were also examined.

**Results:**

Pancreatic cancer cells showed higher expression and activity of GSK3β than non-neoplastic cells, which were associated with changes in its differential phosphorylation. Inhibition of GSK3β significantly reduced the proliferation and survival of cancer cells, sensitized them to gemcitabine and ionizing radiation, and attenuated their migration and invasion. These effects were associated with decreases in cyclin D1 expression and Rb phosphorylation. Inhibition of GSK3β also altered the subcellular localization of Rac1 and F-actin and the cellular microarchitecture, including lamellipodia. Coincident with these changes were the reduced secretion of matrix metalloproteinase-2 (MMP-2) and decreased phosphorylation of focal adhesion kinase (FAK). The effects of GSK3β inhibition on tumor invasion, susceptibility to gemcitabine, MMP-2 expression and FAK phosphorylation were observed in tumor xenografts.

**Conclusion:**

The targeting of GSK3β represents an effective strategy to overcome the dual challenges of invasiveness and treatment resistance in pancreatic cancer.

## Introduction

Pancreatic cancer is a major health problem due to an overall 5-year survival rate of less than 10% [1]. It is characterized by the highly proliferative and invasive capacity of the tumor cells and a strong predisposition for metastasis [2]–[4]. The aggressive nature of pancreatic cancer hampers early diagnosis and curative surgical intervention and renders it resistant to chemotherapy and radiation [3], [4]. The widely used therapy is infusional gemcitabine, although fewer than 20% of patients respond to this treatment [3], [4]. Novel therapeutic strategies that enhance the effects of gemcitabine and attenuate the invasive properties of pancreatic cancer cells are needed. Molecular target-directed therapy has emerged and includes targeting of the growth factor receptors, angiogenic factor/receptor and matrix metalloproteinases, since these are aberrantly expressed in pancreatic cancer [2]–[4]. Several clinical trials of pancreatic cancer have already targeted these growth factors, either as monotherapy or in combination with gemcitabine, but most have shown little or no therapeutic benefit [5]. Identification of novel molecular targets that could enhance the therapeutic effects of gemcitabine and radiation is therefore a high priority [6].

Glycogen synthase kinase 3β (GSK3β) is a serine/threonine protein kinase that regulates multiple signaling pathways [7]. Based on its known functions and involvement in primary pathologies, GSK3β has been implicated as a therapeutic target for glucose intolerance, neurodegenerative disorders and inflammation [8]. We previously demonstrated that deregulated expression, activity and phosphorylation of GSK3β are distinct features of gastrointestinal cancers and glioblastoma and that GSK3β sustains the survival and proliferation of these tumor cells. A role for aberrant GSK3β in these tumor types is supported by the observation that pharmacological inhibition of its activity reduces the survival and proliferation of cancer cells and predisposes them to apoptosis *in vitro* and in tumor xenografts [9]–[12]. Although its role in cancer is still debated [13], the overall results so far indicate that aberrant expression and activity of GSK3β is a common and fundamental characteristic in a broad spectrum of cancers (reviewed in [14]).

Based on earlier studies that demonstrated involvement of GSK3β in NF-κB-mediated cell survival [15], GSK3β was found to support the survival of pancreatic cancer cells via this pathway [16], [17]. Although GSK3β is a key regulator of cell polarization and migration during physiological processes such as tissue development and wound healing [18], very little is known about its role in the migration and invasion of cancer cells. Here we investigated the potential involvement of GSK3β in the invasive nature of pancreatic cancer and its resistance to gemcitabine and ionizing radiation, the two major obstacles to more effective treatment.

## Materials and Methods

### Ethics Statement

Written informed consent was obtained from all patients with pancreatic cancer before surgery. This study was approved by the Medical Ethics Committee of Kanazawa University.

Animal experiments were conducted according to the Guidelines for the Care and Use of Laboratory Animals in Kanazawa Medical University, and in accordance with national guidelines for animal use in research in Japan (http://www.lifescience.mext.go.jp/policies/pdf/an_material011.pdf). The protocol was approved by the Committee on Animal Experiments of Kanazawa Medical University.

### Cell Lines and Tissue Specimens

Human embryonic kidney cells (HEK-293) and pancreatic cancer cells (PANC-1, MIA PaCa-2, BxPC-3, Capan-1) were obtained from the American Type Culture Collection (ATCC). These cell lines were characterized by DNA profiling in ATCC, and passaged for fewer than 6 month after resuscitation. They were maintained at 37°C with 5% CO_2_ in DMEM (HEK-293, PANC-1, MIA PaCa-2, Capan-1) and RPMI 1640 (BxPC-3) supplemented with 10% fetal bovine serum (FBS) and antibiotics (penicillin G and streptomycin) (GIBCO). Cells were harvested during the exponential growth phase for extraction of RNA and protein.

This study included 15 patients with pancreatic cancer who underwent surgery at the Department of Surgical Oncology, Kanazawa University Hospital (Table S1). The surgical specimens were fixed in neutral-buffered 10% formalin, embedded in paraffin and processed for histopathologic diagnosis and immunohistochemical examinations.

### Western Blotting

Protein was extracted from cultured cells using lysis buffer (CelLytic-MT, Sigma-Aldrich) containing a mixture of protease and phosphatase inhibitors (Sigma-Aldrich). Concentrations of protein extracts were measured by Coomassie Protein Assay Reagents (Pierce). A 30 µg aliquot of protein was separated in sodium dodecyl sulfate-polyacrylamide gel electrophoresis (SDS-PAGE) and analyzed by Western blotting for the proteins of interest and their phosphorylation [9], [11], [12] using the respective primary antibodies (Table S2). Electroblotted membranes (Amersham) were blocked with 5% bovine serum albumin prior to detection of phosphorylated protein fractions. The amount of protein in each sample was monitored by the expression of β-actin. Signals were developed using enhanced chemiluminescence (ECL).

### *In vitro* Kinase Assay for GSK3β Activity

A nonradioisotopic *in vitro* kinase assay (NRIKA) [19] was used to detect the activity of GSK3β derived from cells. The NRIKA uses a sequential combination of immunoprecipitations to isolate GSK3β in cellular protein samples, an *in vitro* kinase reaction that uses recombinant human β-catenin protein (substrate) and non-radioisotopic adenosine triphosphate (ATP), followed by immunoblotting to detect the β-catenin phosporylated at serine (S) 33, S37 and/or threonine (T) 41 residues (p-β-catenin^S33/37/T41^) [19]. As a negative control, the mouse monoclonal antibody to GSK3β was replaced by an equal amount of non-immune mouse IgG (Sigma-Aldrich) in the immunoprecipitation step. GSK3β activity is demonstrated by the presence of p-β-catenin^S33/37/T41^ in the test reaction and by its absence in the negative control reaction. The amount of immunoprecipitated GSK3β and the presence of recombinant β-catenin protein in the kinase reaction were monitored by immunoblotting.

### Immunohistochemistry

Expression and/or phosphorylation of GSK3β, matrix metalloproteinase (MMP)-2 and focal adhesion kinase (FAK) in tumor and adjacent non-neoplastic tissues of pancreatic cancer patients were examined by the avidin-biotin-peroxidase complex method [11], [12]. Following deparaffinization, microwave antigen unmasking and blocking of non-specific immunoreactions, paraffin sections were incubated with antibody to GSK3β, tyrosine (Y) 216-phosphorylated GSK3β (p-GSK3β^Y216^), MMP-2, FAK, Y397-phosphorylated or Y861-phosphorylated FAK (p-FAK^Y397^, p-FAK^Y861^) and p-β-catenin^S33/37/T41^, respectively. Sources and working dilutions of these antibodies were shown in Table S2. Sections were then incubated with biotinylated goat anti-rabbit IgG or horse anti-mouse IgG (diluted 1∶200; Vector). Immunoreactivity was detected using the ABComplex/HRP kit (DakoCytomation). For the negative control, primary antibodies were replaced by non-immune rabbit or mouse IgG (DakoCytomation). Overexpression or higher phosphorylation of the respective molecules in tumors was defined as stronger expression or phosphorylation of either protein in the tumor cells compared to non-neoplastic pancreatic ducts in the same patient.

### Effects of GSK3β Inhibition on Cell Survival and Proliferation

Cells seeded in 96-well plates were treated with dimethyl sulfoxide (DMSO; Sigma-Aldrich) or a GSK3β inhibitor (AR-A014418; Calbiochem) dissolved in DMSO at the indicated final concentrations in the medium. Notably, AR-A014418 does not inhibit the activity of 26 closely related kinases and is considered highly specific for GSK3β [20]. At designated time points, the relative numbers of viable and proliferating cells were determined by using WST-8 (4-[3-(4-iodophenyl)-2-(4-nitrophenyl)-2H-5-tetrazolio]-1,3-benzene disulfonate) assay kit (Cell counting kit-8; Wako) and Cell Proliferation ELISA BrdU Kit (Roche), respectively.

Small interfering RNA (siRNA) specific to human GSK3β (GSK3β Validated Stealth RNAi) and negative control siRNA (Stealth RNAi Negative Control Low GC duplex) were purchased from Invitrogen. The GSK3β-specific siRNA targets the sequence 5′-GCUCCAGAUCAUGAGAAAGCUAGAU-3′. Cells were transfected with 10 nM of either siRNA using Lipofectamine RNAiMAX (Invitrogen). The effect of RNA interference on GSK3β expression was determined by Western blotting using an antibody against both GSK3α and β (Table S2). The specificity of GSK3β-specific siRNA was confirmed in our previous studies [11], [12]. To examine the effect of GSK3β RNA interference on cell survival and proliferation, cells seeded into 96-well plates were transfected with 10 nM of control or GSK3β-specific siRNA. At 72 hrs after transfection, the relative numbers of viable and proliferating cells were determined by the methods described above.

### Effects of GSK3β Inhibition on the Susceptibility of Cancer Cells to Gemcitabine and to Ionizing Radiation

Cancer cells seeded into 96-well plates (3–6×10^4^ cells per well) were treated with escalating concentrations of either gemcitabine (Eli Lily) or AR-A014418. Following treatment for 72 hrs, the relative number of viable cells was measured by WST-8 assay to determine IC_50_ (50% cell survival inhibitory concentration) for gemcitabine and AR-A014418 and to generate isobolograms. Cells were then treated with a dose of gemcitabine close to IC_50_ in the presence of DMSO or a low dose (5 or 10 µM) of AR-A014418. The isobologram method [21] was used to determine whether the effect of GSK3β inhibitor on pancreatic cancer cell susceptibility to gemcitabine was additive, synergistic or antagonistic.

The effect of GSK3β inhibitor on pancreatic cancer cell susceptibility to ionizing radiation was examined by colony-forming cell survival assay. In each well of 6-well plates, 1,000 pancreatic cancer cells were seeded and treated sequentially with either DMSO or a low dose (5 or 10 µM) of AR-A014418 for 24 hrs and with ionizing radiation at doses of 0, 4 and 8 Gy. At 6 days after irradiation, the total number of colonies stained with 0.1% crystal violet (Wako) was scored in each well. The mean number of colonies in three separate experiments was calculated with standard deviations.

### Assays for Cell Migration and Invasion

Cancer cell migration and invasion were examined by monolayer-based wound-healing assay and transwell assay. Confluent monolayers of pancreatic cancer cells in the presence of DMSO or AR-A014418 at a dose of 5 or 10 µM were scratched with a 20 µL-micropipette tip to create a cell-free zone (wound). In each condition, the gap distance between the wound edges was monitored at three fixed reference points for 6 to 24 hrs by a CCD camera (Axiocam MRm, Zeiss) connected to a phase-contrast microscope (Axiovert 40 CFL, Zeiss). Cell migration at each time point was calculated as the mean distance of wound measured at the three points and compared between cells treated with DMSO and AR-A014418.

Cell migration and invasion were examined by the transwell assays using uncoated and matrigel-coated 24-well double-chamber system (BD BioCoat™ Matrigel™ Incubation Chamber, BD Bioscience). Cancer cells were suspended in serum-free medium containing DMSO or AR-A014418 (5 or 10 µM), and 2×10^4^ cells were applied to the upper chamber pairing with the lower chamber filled with medium containing 10% FBS (as a chemo-attractant) and DMSO or AR-A014418 (5 or 10 µM). At 22 hrs after allowing cells to migrate and invade, cells on the lower side of the chamber were fixed and stained with Diff-Quick Kit (Symex). In each assay, the total number of cells per high-power microscopic field on the lower side of the uncoated or matrigel-coated chamber was counted and scored for migrating or invading cells. The mean number of cells in five high-power microscopic fields was calculated with standard deviations.

### Cell Morphology and Immunofluorescence Cytochemistry

Cancer cells grown on a cover slip were treated with either DMSO or AR-A014418 (5 or 10 µM) for 12 hrs and then scratched as described above. At 12 hrs after scratching, the cells along the wound edges were observed by phase-contrast microscopy. These cells were then fixed with 4% paraformaldehyde and permeabilized with 0.1% Triton-X (Sigma-Aldrich). The cells were incubated serially with mouse monoclonal antibody to Rac1 (BD Bioscience; diluted 1∶200) at 4°C overnight and with Alexa Flour® 488-labeled anti-mouse IgG (Invitrogen; diluted 1∶1,000) at room temperature for 40 min in the dark. After washing off excess antibody, the cells were stained for filamentous (F-) actin with Alexa Flour® 546-labeled phalloidin (Invitrogen; diluted 1∶40) for 20 min. Then, cell nuclei were counterstained with Hoechst 33342 (Molecular Probes) and observed by fluorescence microscopy (Keyence) for expression and subcellular localization of Rac1 and F-actin.

### Rac1 Activity

Protein was extracted from cells treated with DMSO or 10 µM AR-A014418 for 24 hrs in 25 mM Tris-HCl buffer (pH 7.5) containing 150 mM NaCl, 5 mM MgCl_2_, 1% NP-40, 1 mM dithiothreitol and 5% glycerol. Active Rac1 was isolated from the protein sample by the pull-down method using GST-human Pak1-PBD (Thermo) and resins (Glutathione Sepharose 4 Fast Flow; GE Healthcare). The fraction of Rac1 bound to guanosine triphosphate (GTP) (Rac1-GTP, an active form) was eluted from the resins and detected by Western blot analysis using rabbit polyclonal antibody to Rac1 (diluted 1∶1,000; Thermo). Separately, whole cellular protein was probed for total Rac1 using the same antibody.

### Expression and Secretion of Matrix Metalloproteinase-2 (MMP-2)

Expression of MMP-2 mRNA was examined by quantitative reverse transcription-PCR (qRT-PCR). Total RNA was isolated from cells using ISOGEN (Wako). Complementary DNA (cDNA) was generated using a Reverse Transcription Kit (Promega). qRT-PCR was performed using SYBR Premix Ex Taq^TM^II (Takara Bio) with the respective sets of sense and antisense primers for amplification of MMP-2 and β-actin (Table S2) [11].

MMP-2 expression was analyzed by gelatin zymography [22]. Cancer cells were seeded on 12-well plates for 48 hrs and then treated with DMSO or AR-A01418 (10 or 25 µM) for 24 hrs in serum-free medium. Conditioned medium or treated cells were incubated with SDS sample buffer for 30 min at 37°C. Samples were separated on 10% SDS-PAGE containing 0.005% Alexa Fluor 680-labelled gelatin. After electrophoresis, gels were washed in 2.5% Triton X-100 for 2 hrs and then incubated in substrate buffer overnight at 37°C. The gel was scanned by the LI-COR Odyssey IR imaging system (Lincoln).

### Tumor Xenograft Study

We prepared subcutaneous PANC-1 xenografts in mice as described [23]. These mice were assigned to 4 groups and treated with intraperitoneal injection (twice a week for 10 weeks) of DMSO (diluent), gemcitabine (20 mg/kg body weight) and AR-A014418 (2 mg/kg body weight, equivalent to 10 µM in culture medium as determined and optimized in our previous studies [10], [12]) alone or in combination, respectively. All mice were terminated after treatment and the xenograft tumors were removed and processed for histological examination and immunohistochemistry for expression of MMP-2 and FAK and phosphorylation of FAK in tumor cells, as described above.

### Statistical Analysis

Between-group statistical significance was determined using the Student *t* test. In tumor xenograft study, tumor volumes in each treatment group were expressed as means ± standard deviation (SD). The statistical significance of differences among the data was determined with Kruskal Wallis H-test followed by Mann-Whitney U-test with Bonferroni correction. A *P* value of <0.05 was considered statistically significant.

## Results

### Expression, Phosphorylation and Activity of GSK3β in Cancer Cells

**Figure 1:**
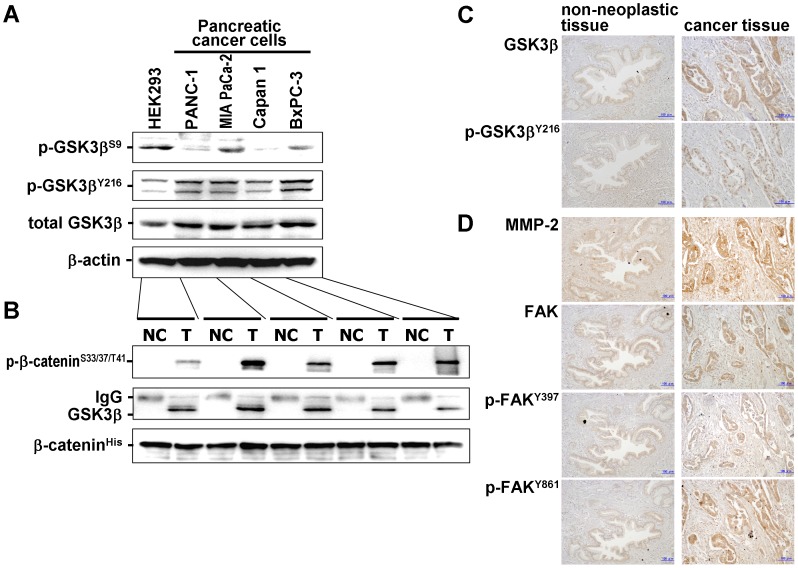
Expression, phosphorylation and activity of GSK3β in pancreatic cancer cells and primary pancreatic cancers. (A) Protein extract from each cell line was analyzed by Western immunoblotting for the expression of GSK3β and its phosphorylation (p-GSK3β^S9^, p-GSK3β^Y216^). β-actin expression was monitored as a loading control. (B) GSK3β activity was detected by NRIKA [19] in the respective cells. As described in Materials and Methods, GSK3β activity is demonstrated by the presence of p-β-catenin^S33/37/T41^ in the test reaction (T) and by its absence in the negative control reaction (NC). The amount of immunoprecipitated GSK3β and the presence of substrate (β-catenin) in the kinase reaction were monitored by immunoblotting. (C, D) Serial paraffin sections of a primary pancreatic cancer and its adjacent non-neoplastic tissue (patient No. 5 in Table S1) were immunostained for GSK3β and p-GSK3β^Y216^ (C), and for MMP-2, FAK, p-FAK^Y397^ and p-FAK^Y861^ (D). The scale bar in each panel indicates 100-µm in length.

Pancreatic cancer cells showed higher basal levels of GSK3β and the Y216-phosphorylated active form (p-GSK3β^Y216^) and lower levels of the S9-phosphorylated inactive form (p-GSK3β^S9^) compared to HEK 293 cells (Fig. 1A). Cancer cell-derived GSK3β was active for phosphorylation of its substrate, β-catenin (Fig. 1B). These results indicate that pancreatic cancer cells express active GSK3β that is not regulated by differential phosphorylation at S9 and Y216. Immunohistochemistry for the serial sections showed that GSK3β and p-GSK3β^Y216^ were diffusely expressed and colocalized in the tumor cells and overexpressed in the invasive tumor cells of 8/15 (53%) pancreatic cancer patients (Fig. 1C). Overexpression was more frequent in patients with T3/T4 primary tumor or with lymph node and distant metastasis at the time of surgery (Table S1).

### Effects of GSK3β Inhibitor on Cell Survival and Proliferation

**Figure 2:**
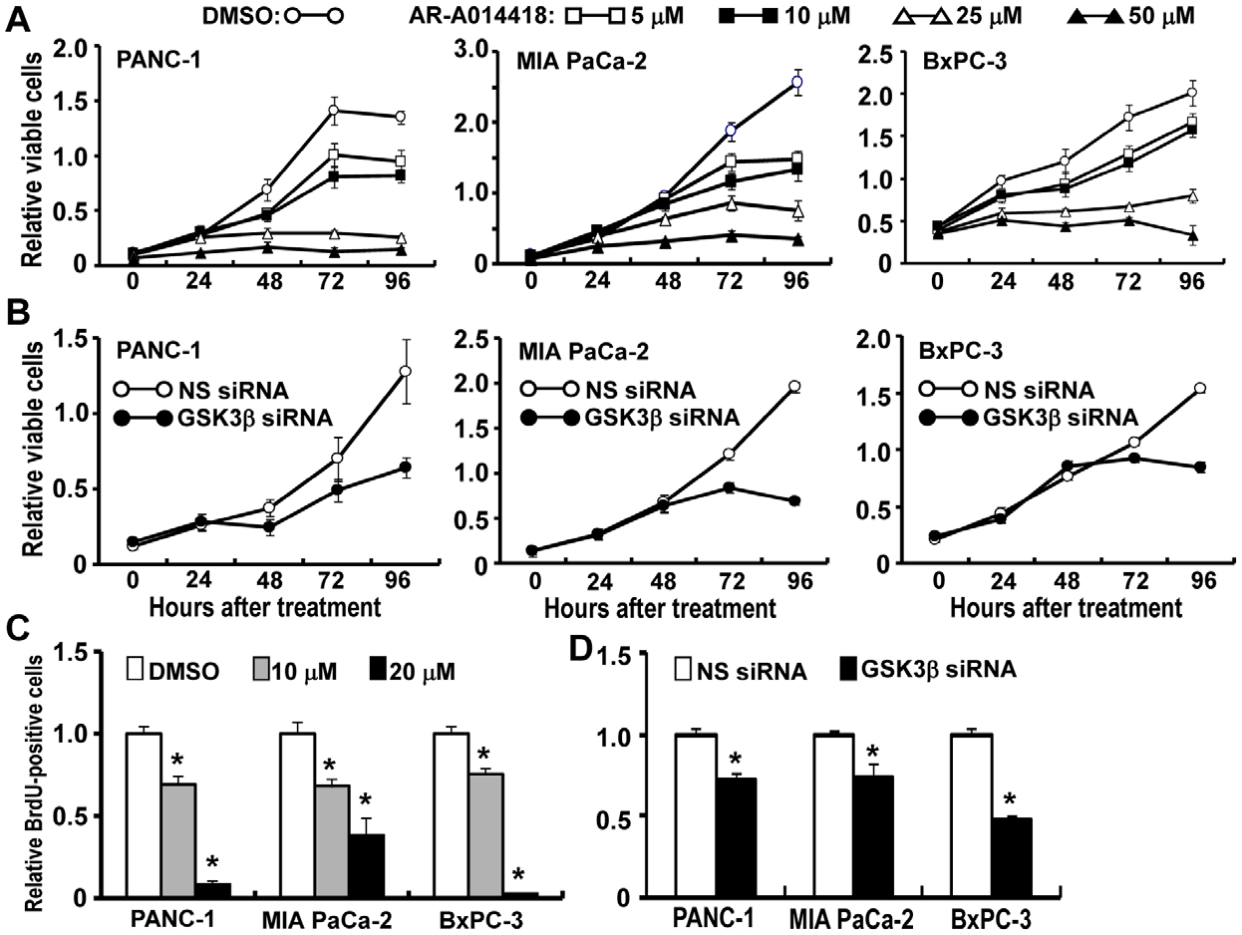
Effects of GSK3β inhibition on the survival and proliferation of pancreatic cancer cells. (A) Relative numbers of viable cells at the designated time points were measured by WST-8 assay for the respective cells in the presence of DMSO or AR-A014418 at the indicated concentrations. (B) Relative numbers of viable cells were measured for the respective cells after transfection of non-specific (NS) or GSK3β-specific siRNA. (C, D) The relative number of proliferating cells was determined by measuring the amount of BrdU incorporation. Proliferating cells were scored at 48 hrs after treatment with DMSO or AR-A014418 (10 µM, 20 µM) (C), or after transfection with non-specific (NS) or GSK3β-specific siRNA (D). Values shown in (A–D) are the means ± SD of five separate experiments. *p<0.05, statistically significant difference between cells treated with DMSO or AR-A014418 and between cells treated with non-specific and GSK3β-specific siRNA.

We investigated whether GSK3β contributes to cancer cell survival and proliferation. The levels of glycogen synthase (GS) phosphorylated at Y641 (p-GS^S641^) and p-β-catenin^S33/37/T41^ decreased in cancer cells following treatment with AR-A014418 (Fig. S1A, B), indicating its activity against GSK3β in cancer cells. GSK3β inhibition attenuated the survival and proliferation of cancer cells (Fig. 2A, C). Depletion of GSK3β by RNA interference attenuated cell viability and proliferation in all cancer cell lines (Fig. 2B, D). These results are consistent with the previous studies [16], [17] suggesting that aberrant GSK3β impacts upon the survival and proliferation of pancreatic cancer cells.

### Effects of GSK3β Inhibitor Combined with Gemcitabine or Radiation Against Cancer Cells

**Figure 3:**
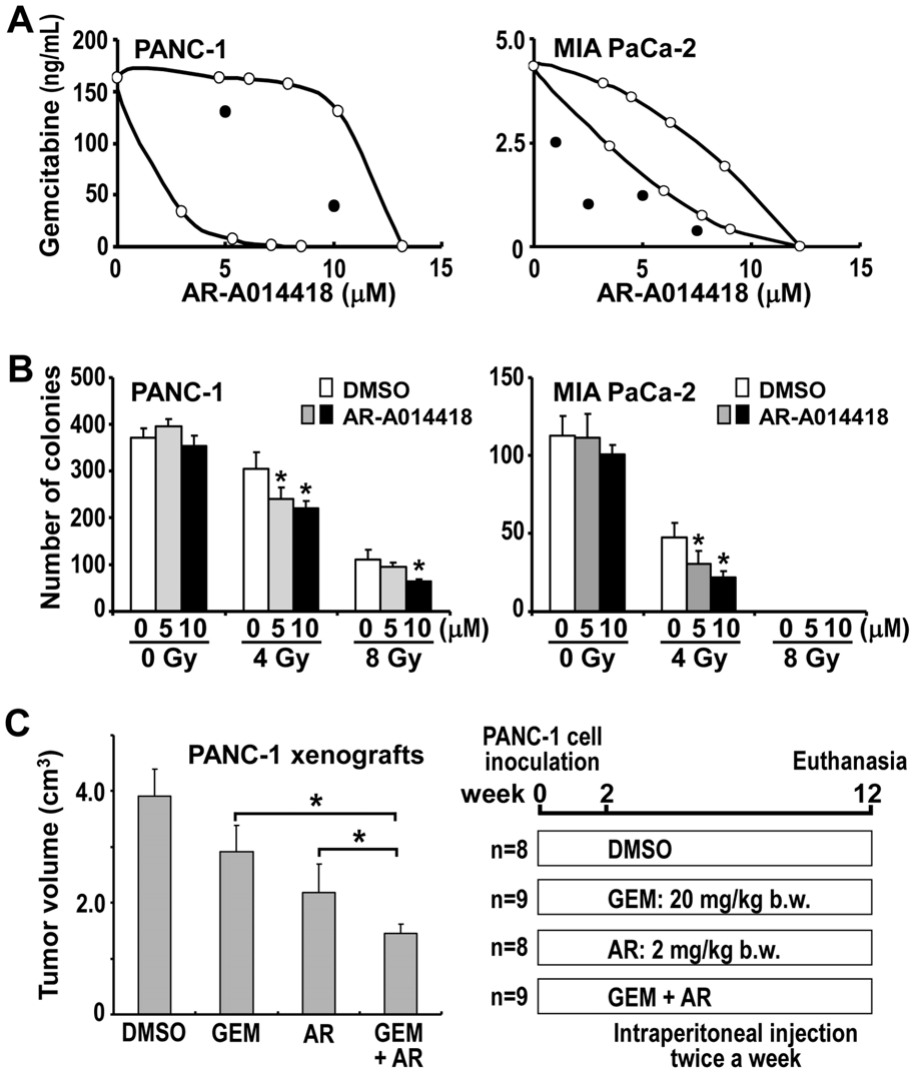
Combined effect of gemcitabine or ionizong radiation and GSK3β inhibitor against cancer cells and xenografts. (A) The influence of AR-A014418 on the effect of gemcitabine was analyzed using the isobologram [21] by plotting the IC_50_ of the combination therapy (Fig. S2, Table S4). (B) The combined effect of ionizing radiation and AR-A014418 was tested in PANC-1 and MIA PaCa-2 cells by colony formation assay. *p<0.05, statistically significant difference between cells treated with DMSO or AR-A014418. (C) The combined effect of gemcitabine and AR-A014418 was tested in PANC-1 xenografts. Athymic mice with PANC-1 xenograft were assigned to four groups for treatment with intraperitoneal injection (twice a week) of DMSO (control; 8 mice), gemcitabine (GEM; 20 mg/kg body weight; 9 mice) and AR-A014418 (AR; 2 mg/kg body weight; 8 mice), alone or in combination (GEM+AR; 9 mice). At the time after treatment for 10 weeks, tumor volume (cm^3^) was calculated using the formula 0.5×S^2^×L, where S is the smallest tumor diameter (cm) and L is the largest (cm) [10], [12]. The mean tumor volume was compared between the 4 groups. *p<0.05, statistically significant difference between data.

The above results led us to address whether GSK3β inhibition could enhance the effects of gemcitabine and ionizing radiation. High doses (25 or 50 µM) of AR-A014418 alone had a therapeutic effect against cancer cells (Fig. 2A, C). Therefore, the effects of relatively low doses (5 or 10 µM) were tested in combination with gemcitabine or ionizing radiation. First, we examined dose-dependent effects of AR-A014418 and gemcitabine on cancer cell survival and determined their IC_50_ values (Fig. 2A, Fig. S2). IC_50_ values for AR-A014418 were similar in PANC-1, MIA PaCa-2 and BxPC-3 cells, whereas those for gemcitabine varied (Table S3). We next examined the effect of AR-A014418 on the susceptibility of cancer cells to gemcitabine. When cells were treated with escalating doses (1 ng/mL to 10 µg/mL) of gemcitabine, combination with low dose AR-A014418 significantly reduced the IC_50_ of gemcitabine (Fig. S2, Table S4). Isobologram analysis [21] of the data revealed that low-dose AR-A014418 in combination with gemcitabine was additive against PANC-1 cells and synergistic against MIA PaCa-2 cells (Fig. 3A). We confirmed that the combined treatment with AR-A014418 significantly enhanced the effect of gemcitabine against cancer cell xenografts (Fig. 3C) in rodents with no detrimental effects by the reagent.

The effect of AR-A014418 combined with ionizing radiation was tested in cancer cells. In colony-forming cell survival assay, presence of 10 µM AR-A014418 significantly reduced viability of the cancer cells compared to treatment with ionizing radiation alone (Fig. 3B). Together, these results demonstrate that combined treatment with GSK3β inhibitor sensitizes cancer cells to gemcitabine and to ionizing radiation.

### Molecular Alterations Associated with GSK3β Inhibition in Cancer Cells

**Figure 4:**
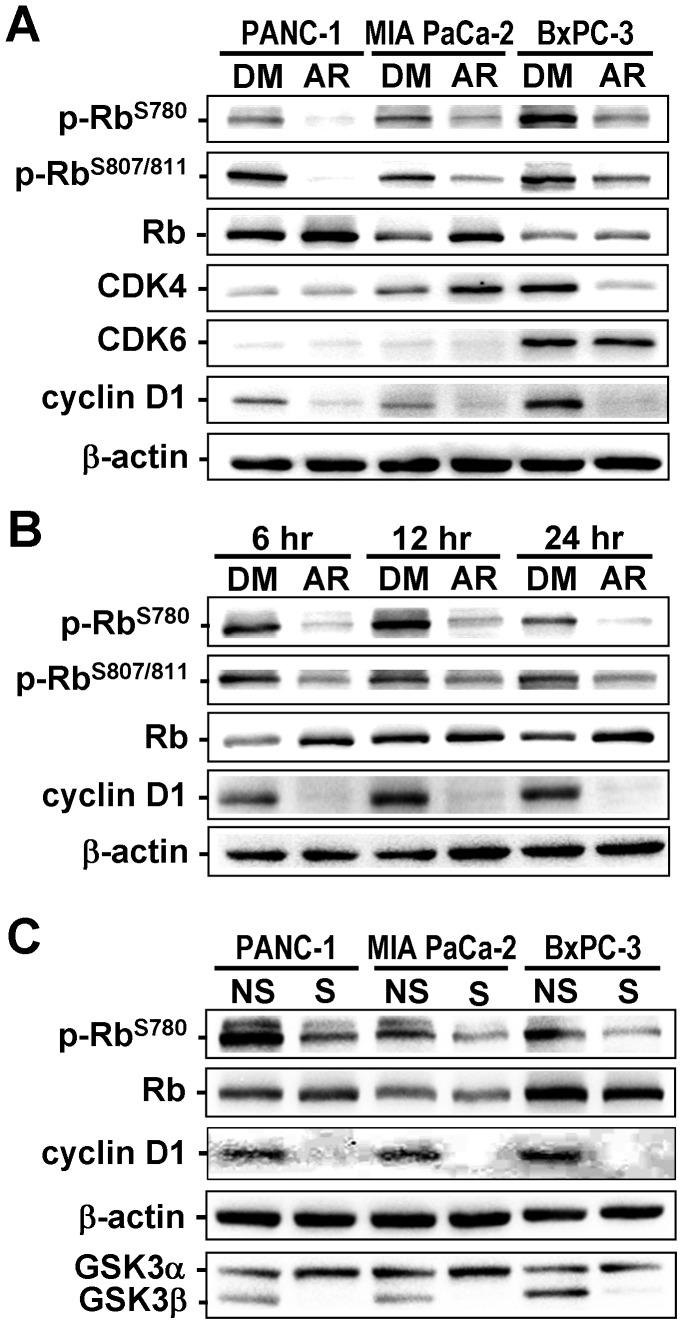
Changes in expression and phosphorylation of the proteins in cancer cells following GSK3β inhibition. (A) Immunoblotting analysis compares the expression of Rb, CDK4, CDK6 and cyclin D1, and the phosphorylation of Rb at S780 and S807/811 residues (p-Rb^S780^, p-Rb^S807/811^) between cells treated with DMSO (DM) or 10 µM AR-A014418 (AR) for 24 hrs. (B) Changes in levels of p-Rb^S780^ and p-Rb^S807/811^ and expression of Rb and cyclin D1 were examined in MIA PaCa-2 cells at the indicated time points after treatment with 10 µM AR-A014418. (C) Expression of Rb, cyclin D1, GSK3α and GSK3β proteins and levels of Rb phosphorylation (p-Rb^S780^) were examined and compared between the same pancreatic cancer cells transfected with non-specific siRNA (NS) or GSK3β-specific siRNA (S) (10 nM each). (A–C) β-actin expression was monitored as a loading control.

To understand the mechanism that underlies involvement of GSK3β in cancer cell proliferation and resistance to therapy, we investigated the effect of GSK3β inhibition on expression and phosphorylation of proteins involved in cell cycle regulation and proliferation. Consistent with previous studies (reviewed in [2]), pancreatic cancer cells showed phosphorylation of Rb protein (p-Rb^S780^, p-Rb^S807/811^; Fig. 4), suggesting that binding to the E2F transcription factor was impaired [24]. Treatment with either AR-A014418 or GSK3β-specific siRNA decreased the levels of Rb phosphorylation and cyclin D1 expression (Fig. 4), however no consistent changes were found for CDK4 or CDK6 expression.

### Effects of GSK3β Inhibition on Cancer Cell Migration and Invasion

**Figure 5:**
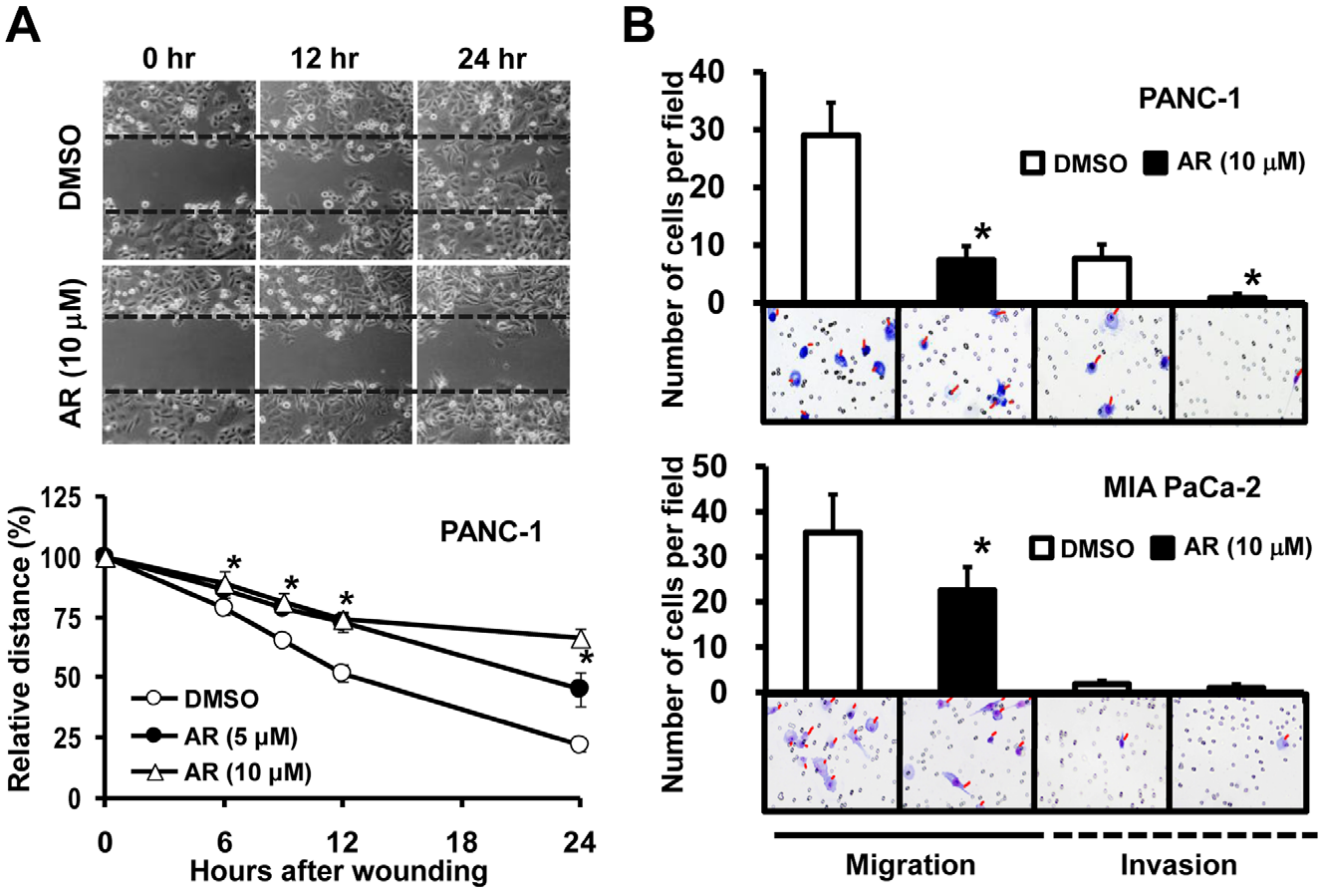
Effects of GSK3β inhibition on the migration and invasion of pancreatic cancer cells. (A) Upper panels show the time course for PANC-1 cell migration in a wound-healing assay in the presence of DMSO or AR-A014418 (AR). The lower panel shows the relative widths of wounds measured as a percentage of the initial gap at time zero. *p<0.05, statistically significant difference between cells treated with DMSO or AR-A014418. (B) Migrating cells through uncoated transwell and invading cells through matrigel-coated transwell were scored for PANC-1 and MIA PaCa-2 cells treated with DMSO or AR-A014418 (AR) for 22 hrs. Representative photomicroscopic findings in each assay are shown below the columns. *p<0.05, statistically significant difference between cells treated with DMSO or AR-A014418.

The wound-healing assay showed that migration of cancer cells was significantly reduced by treatment with 5 µM and 10 µM AR-A014418 (Fig. 5A, Fig. S3). Importantly, these concentrations were insufficient to inhibit cell proliferation 24 hrs after treatment (Fig. 2A). In the transwell assay, 5 µM and 10 µM AR-A014418 inhibited chemotactic migration of cancer cells and their invasion of extracellular matrix component (Fig. 5B).

### Changes in the Invasive Phenotype of Cancer Cells Following GSK3β Inhibition

The above results led us to hypothesize that GSK3β has a role in cancer cell migration and invasion. This may be attributed to epithelial-mesenchymal transition (EMT), a phenotype responsible for cancer cell invasion and metastasis [25]. We investigated expression of the EMT-related molecules E-cadherin, N-cadherin and vimentin in cancer cells following treatment with AR-A04418 and GSK3β-specific siRNA. No consistent changes were observed in the levels of expression of these molecules (Fig. S1C, D), implying that EMT is unlikely to be the mechanism by which GSK3β inhibition attenuates cancer cell migration and invasion. A possible explanation may be that established cancer cell lines have already acquired the EMT phenotype.

**Figure 6:**
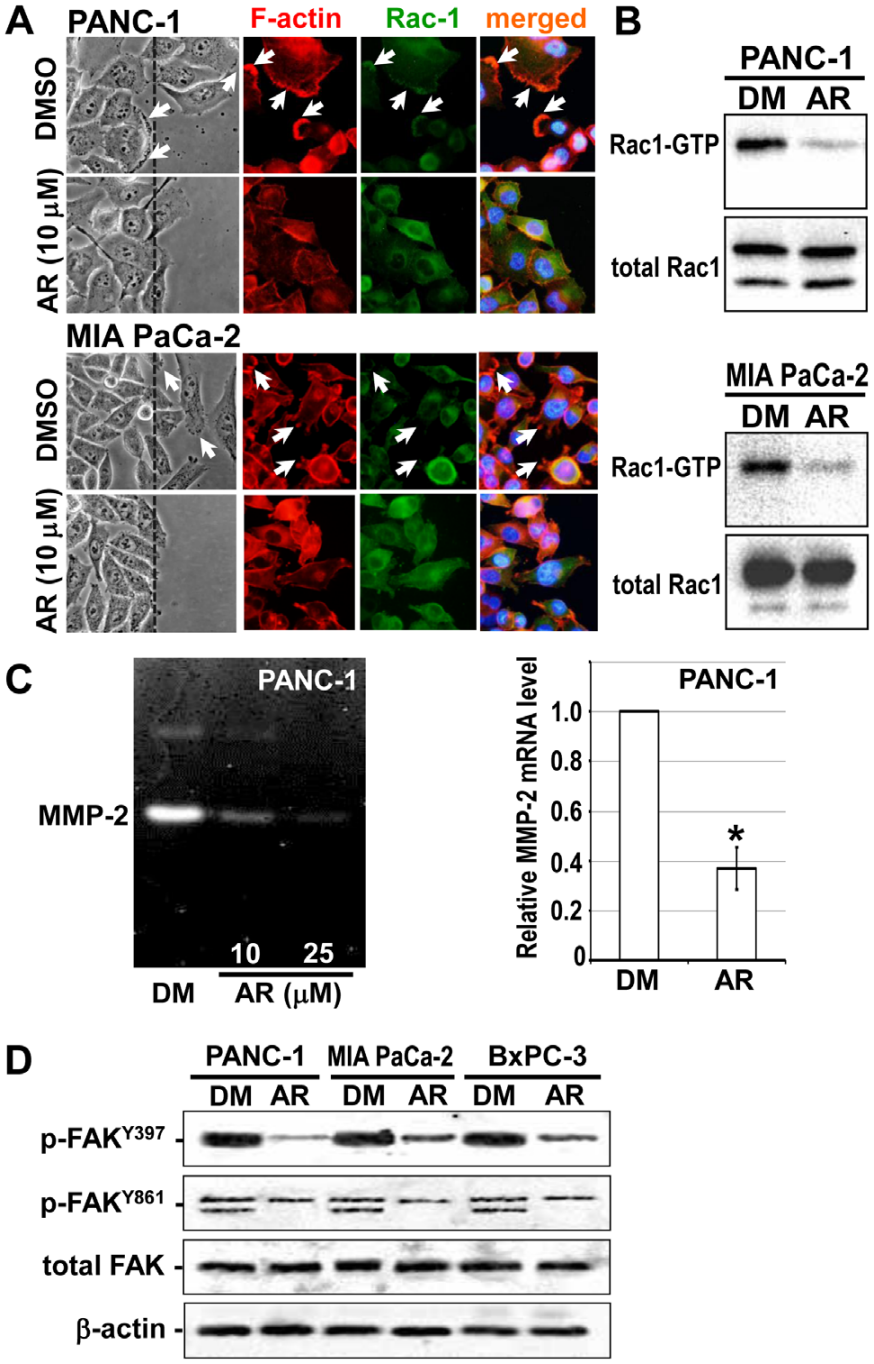
Changes in the invasive phenotype of pancreatic cancer cells following GSK3β inhibition. (A) Phase-contrast microscopic findings (left panels), expression and subcellular localization of F-actin and Rac-1 (middle panels) and their merged images (right panels) in cancer cells along the wound edge (dashed line) were observed in the wound-healing assay in the presence of DMSO or AR-A014418 (AR). Arrows indicate lamellipodia. (B) Changes in the levels of active (Rac1-GTP) and total Rac1 examined by pull-down assay and Western blotting between the cancer cells treated with DMSO (DM) or 10 µM AR-A014418 (AR) for 24 hrs. (C) Changes in the secretion and mRNA expression of MMP-2 examined by gelatin zymography (left panel) and qRT-PCR (right panel) between PANC-1 cells treated with DMSO (DM) or AR-A014418 (AR) for 24 hrs. Values for the relative levels of mRNA expression are shown as means ± SDs of four separate experiments. *p<0.05, statistically significant difference between cells treated with DMSO or AR-A014418. (D) Changes in the expression of FAK and its phosphorylation (p-FAK^Y397^, p-FAK^Y861^) examined by Western blotting in cancer cells following GSK3β inhibition. The cells in confluent monolayer were wounded multiple times and cultured in the presence of DMSO (DM) or 10 µM AR-A014418 (AR) for 24 hrs. β-actin expression was monitored as a loading control.

We next focused on cellular microarchitecture and in particular on lamellipodia that plays an important role in cell migration during physiological processes and in cancer cell migration and invasion [26]. A member of the Rho-GTPase family, Rac1, participates in lamellipodia formation and in cancer progression [27]. Wound healing assay showed that migrating cancer cells form lamellipodia at the site of Rac1 localization, with actin filaments organizing the lamella structure. Treatment with AR-A014418 decreased lamellipodia formation in cancer cells at the wound edge and resulted in diffuse cytoplasmic distribution of Rac1 and F-actin (Fig. 6A). Concomitant with these changes, treatment with AR-A014418 decreased the Rac1-GTP (active form) in cancer cells stimulated to multiply by the regenerating wounds (Fig. 6B).

Rac1 was reported to increase the secretion and activity of MMP-2 in cancer cells [28]. Treatment with AR-A014418 inhibited MMP-2 secretion and decreased MMP-2 mRNA expression (Fig. 6C). These results indicate a mechanistic link between GSK3β and Rac1 in the regulation of MMP-2 expression and secretion. Previous studies have reported that FAK regulates Rac1 [29]. We therefore investigated the effect of GSK3β inhibition on FAK activity in cancer cells responding to wound stimulation by determining the levels of p-FAK^Y397^ and p-FAK^Y861^ [29]. FAK phosphorylation was detected in cancer cells stimulated by multiple wounds. Treatment with 10 µM AR-A014418 reduced the levels of p-FAK^Y397^ and p-FAK^Y861^ (Fig. 6D), suggesting a pivotal role for the GSK3β/FAK/Rac1 pathway in promoting pancreatic cancer cell invasion.

**Figure 7:**
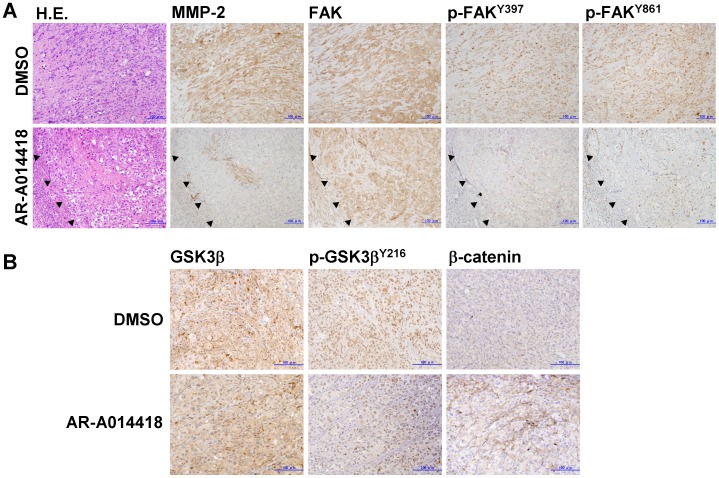
Changes in the invasive phenotype of pancreatic cancer cells in xenografts following GSK3β inhibition. (A) The left two panels showed represntative histological findings of the deeper part of PANC-1 xenografts in rodents after treatment with intraperitoneal injection (twice a week) of DMSO and AR-A014418, respectively, as shown in Fig. 3C. The serial sections of these tumors were immunostained for MMP-2, FAK, p-FAK^Y397^ and p-FAK^Y861^. Closed triangles in the lower panels delineate an interface between the xenograft and host stromal tissue. (B) These tumors were immunostained for GSK3β, p-GSK3β^Y216^ and β-catenin. (A, B) The scale bar in each panel indicates 100-µm in length.

Immunohistochemical examination of the primary pancreatic cancers showed higher expression of MMP-2 and FAK and tyrosine phosphorylation of FAK in tumor cells than non-neoplastic pancreatic ducts (Fig. 1D). In histological examination of the deep part of cancer cell xenografts, the tumor in mice treated with AR-A014418 showed less invasive than those in sham (DMSO)-treated mice (Fig. 7A, left panels). Immunohistochemistry showed decreases in MMP-2 expression and FAK phosphorylation in the tumors by treatment with AR-A014418 (Fig. 7A). Both groups of mice showed the similar levels of GSK3β expression in tumor xenografts (Fig. 7B, left panels). In AR-A014418-treated mice, Y216-phosphorylation of GSK3β appeared to decrease in the tumors, and membranous expression, but no nuclear accumulation of β-catenin was observed in tumor cells (Fig. 7B).

## Discussion

Failure of treatments for pancreatic cancer is due to the high propensity of these tumors to invade surrounding tissues and because of their resistance to chemotherapy and radiation [3], [4]. The present study confirmed previously reported roles for GSK3β in cancer cell survival and proliferation [16], [17]. However, the novel findings of this study relate to the effects of GSK3β inhibition on the invasive ability and phenotype of pancreatic cancer cells and on their susceptibility to gemcitabine and radiation. The results provide a biological rationale for combinational treatment strategies that include targeting of GSK3β in order to control refractory pancreatic cancer.

The proinvasive phenotype of cancer cells includes EMT and increased cell motility [25], [26]. Here, we found overexpression of GSK3β and its active form in the tumor cells in the invasive primary pancreatic cancers. Inhibition of GSK3β attenuated cancer cell migration and invasion in wound-healing and transwell assays. No consistent changes were observed for the expression of EMT-related molecules in cancer cells treated with GSK3β inhibitor, in contrast to a previous study that found GSK3β inhibits EMT by phosphorylating and destabilizing snail, a transcriptional repressor of E-cadherin [30]. Our study suggests that EMT may not be involved in the mechanism by which GSK3β inhibition attenuates cancer cell migration and invasion. In wound-healing assays, the migrating cancer cells formed lamellipodia where Rac1 and F-actin preferentially co-localized. Treatment with GSK3β inhibitor decreased lamellipodia formation and re-distributed Rac1 and F-actin throughout the cytoplasm. GSK3β inhibition decreased the active fractions of FAK and Rac1 and the expression and secretion of MMP-2. These findings indicate for the first time the existence of a key pathway involving GSK3β, FAK and Rac1 that plays a pivotal role in promoting pancreatic cancer invasion and could provide a novel target for inhibiting cancer cell invasion.

Rac1 cycles between active GTP-bound and inactive guanosine diphosphate (GDP)-bound forms. Rho-GTPase activity is regulated by three classes of proteins including guanine nucleotide exchange factors (GEFs), GTPase-activating proteins (GAPs) and GDP dissociation inhibitors [27]. GEFs that activate GTPases participate in cancer progression and are considered as therapeutic targets [31]. Aberrant expression of Vav1, a Vav family member of GEFs, is frequently observed in pancreatic cancer and associated with active Rac1 and worse prognosis [32]. Within the cell migration machinery, the IQ motif-containing GAPs (IQGAPs) form scaffolds with Rac1 and Cdc42 (another member of Rho-GTPases) and bind to F-actin. In migrating cells, this molecular complex localizes to actin-rich cellular structures such as lamellipodia and controls F-actin and microtubule dynamics [33]. GSK3β regulates microtubule stability by phosphorylating microtubule-binding proteins, while Cdc42 and GSK3β interact spatially to dictate the polarity of migrating cells [34], [35]. IQGAPs participate in cancer cell invasion and the overexpression of IQGAP1 is associated with unfavorable prognosis in various cancers [36], [37]. A previous study indicated that Rac1 destabilizes E-cadherin-mediated cell adhesion in pancreatic cancer by interacting with IQGAP1, thereby promoting cancer cell migration [38]. Further work is required to clarify the putative roles for GSK3β in regulating cytoskeletal structure, cell polarity and motility and its promotion of cancer cell migration and invasion.

Resistance of cancer cells to chemotherapeutic agents, radiation and molecular target-directed agents is a critical determinant of outcome from recurrent and unresectable pancreatic cancer [3]–[5]. Many studies have investigated the mechanisms by which pancreatic cancer cells resist or acquire resistance to chemotherapy and radiation, focusing mainly on NF-κB [39]. GSK3β was reported to sustain pancreatic cancer cell survival by maintaining the transcriptional activity of NF-κB [16], [17]. However, a recent study found that disruption of NF-κB activity through GSK3β inhibition did not sensitize cancer cells to gemcitabine [40]. Whereas these studies examined the activity of exogenous (transfected) NF-κB, we previously found no effect of GSK3β inhibition on endogenous NF-κB transcriptional activity in gastrointestinal cancers (including pancreatic cancer) and glioblastoma [11], [12]. Therefore, a role for GSK3β in regulating NF-κB activity in cancer cells remains controversial.

In the present study, we tested various combinations and doses of gemcitabine and AR-A014418 on the survival of pancreatic cancer cells. AR-A014418 sensitized the cells to gemcitabine when the doses of both agents were individually optimized. Consistent with our preliminary study [23], the present study confirmed the effect of this drug combination in the cancer cell xenografts. Elucidation of the underlying biological mechanism is important to justify the combinational treatment of GSK3β inhibitor with gemcitabine. One possible mechanism is suggested by our observation that GSK3β inhibition decreased Rb phosphorylation, thus restoring its function against E2F. Ribonucleotide reductase (RR), thymidylate synthase (TS) and thymidine kinase (TK) are transcriptional targets for E2F and essential for DNA synthesis and replication [41]. It was reported that pancreatic cancers with increased RR expression are resistant to gemcitabine [42]. Therefore, restoration of Rb ability to bind to E2F following GSK3β inhibition may sensitizes cancer cells to gemcitabine by affecting RR expression. Inhibition of GSK3β also enhanced the effect of ionizing radiation against cancer cells. Decreases in E2F-dependent transcription of TS and TK may be responsible for this radio-sensitization effect via the impairment of radiation-induced DNA damage repair. In contrast to our result, a recent study reported that siRNA-mediated GSK3β silencing promotes pancreatic cancer cell survival following irradiation via stabilization and activation of β-catenin [43]. Clarification of the role of GSK3β in modulating the anti-tumor effects of radiation is therefore a priority for future investigations.

While the levels of GSK3β activity differ between the pancreatic cancer cells examined in this study (Fig. 1B), its inhibitor AR-A014418 has the similar IC_50_ values against them (Fig. 2A, Table S3). This is consistent with our previous studies showing the similar therapeutic effects of this inhibitor in its pharmacologic doses [20] against different gastrointestinal cancer cells [9], [12] and glioblastoma cells [11]. Other studies also reported the similar result in pancreatic cancer cells [16], [17]. These studies may suggest that survival of different cancer cells and/or their susceptibility to AR-A014418 might similarly depend on GSK3β activity in cells. This issue should be an important future task for cancer treatment targeting GSK3β.

Several studies have suggested opposite roles for GSK3β in the same cellular events mediated by the protooncoproteins and tumor suppressors between non-neoplastic and cancer cells [14]. Whereas GSK3β phosphorylates and stabilizes p27^Kip1^ in normal cells [44], it down-regulates p27^Kip1^ in leukemia cells and selectively maintains the survival and proliferation of these cells [45]. Despite its role in destabilizing cyclin D1 in physiological cells [7], [8], [13], inhibition of GSK3β decreases cyclin D1 expression in cancer cells [11], [12], [46]. Other studies reported various roles for GSK3β in regulating cell stemness. It was shown that GSK3β inhibition maintains the pluripotency of embryonic stem cells and the repopulation of hematopoietic stem cells through activation of the Wnt and hedgehog pathways, respectively [47], [48]. Conversely, it was reported that GSK3β sustains tumor cell stemness in leukemia and glioblastoma [45], [49]. Our present and previous studies showing the presence of both inactive (p-GSK3β^S9^) and active (p-GSK3β^Y216^) forms in non-neoplastic cells and tissues [9], [12] suggest that the kinase activity is regulated by differential phosphorylation in these key residues depending on stimuli in cells of non-neoplastic origin. Although the underlying mechanisms are yet to be understood, differential roles for GSK3β in normal and neoplastic cells could be advantageous for cancer treatment strategies that target this kinase. Consistently, the animal studies showed little detrimental effects of GSK3β inhibition on normal cells and vital organs [10], [12], [23], leading to promotion of future clinical application of cancer treatment targeting GSK3β.

Concerns regarding the therapeutic use of GSK3β inhibitors remain, because these may activate oncogenic (e.g., Wnt) signaling and thus promote cellular transformation [7], [8], [13]. Unlike this hypothesis, inhibition of GSK3β is not sufficient to stabilize β-catenin in normal cells and this seems to occur only when other transforming events (eg., adenomatous polyposis coli [APC] protein truncation) have already taken place [50]. In normal cells, the known function of GSK3β in mediating Wnt/β-catenin signaling depends on cell membrane-associated fraction of GSK3β that antagonizes the phosphorylation of β-catenin by cytoplasmic GSK3β, a key step initiating β-catenin degradation [51]. These paradoxical roles of GSK3β in cells partly supports the reports showing that GSK3β inhibition does not influence the survival or growth of normal cells, nor induce their transformation ([9], [12], [16], [19]; reviewed in [14]). Consistently the above concerns have not deterred preclinical studies of GSK3β inhibitors for the treatment of many cancer types [14], or Phase II clinical trials for the treatment of neurological diseases [52]. These trials are rationally supported by the differential roles of GSK3β in cellular signaling events between normal and tumor cells and by the phosphorylation-dependent regulation of GSK3β activity that presumably protects normal cells from transformation by GSK3β inhibition [9]–[12], [14]. Currently, two clinical trials are being undertaken to test whether the GSK3β inhibitor LY2090314 (Eli Lily) enhances the efficacy of established chemotherapeutic agents for advanced solid cancers (http://clinicaltrials.gov/ct2/show/study/NCT01287520) and leukemia (http://clinicaltrials.gov/ct2/show/study/NCT01214603).

## Supporting Information

Figure S1 **Effects of GSK3β inhibition on expression and phosphorylation of the proteins in pancreatic cancer cells.** The levels of expression and phosphorylation of the indicated proteins were examined by Western blotting in pancreatic cancer cells after treatment with the respective agents. (A, B) Expression of GS and β-catenin and their phosphorylation (p-GS^S641^, p-β-catenin^ S33/37/T41^) were examined and compared between the same pancreatic cancer cells treated with DMSO (DM) or 10 µM AR-A014418 (AR) for 6 hrs. (C) Expression of E-cadherin, N-cadherin and vimentin in pancreatic cancer cells treated with DMSO (DM) or 10 µM AR-A014418 (AR) for 6 hrs. (D) Expression of E-cadherin, N-cadherin, vimentin and GSK3α and GSK3β in pancreatic cancer cells transfected with non-specific siRNA (NS) or GSK3β-specific (S) siRNA (10 nM each). (A–D) The amount of protein extract in each sample was monitored by expression of β-actin.
(TIF)

Figure S2 **Effects of gemciatbine and AR-A014418, alone or in combination, against pancreatic cancer cells.** Inhibitory effects of gemcitabine, AR-A014418 and combinations of the two agents at different doses were examined on the survival of pancreatic cancer cells. PANC-1 (A) and MIA PaCa-2 (B) cells were treated with escalating doses of either gemcitabine, AR-A014418 or both agents in combination at the doses indicated. Relative (%) cell survival ratios for each cell line were examined by WST-8 assay at 48 hrs after treatment with the respective agent. IC_50_ of gemcitabine in the absence (+ DMSO) or presence of AR-A014418 (+ AR) at the indicated doses was determined and is shown in Table S4.
(TIF)

Figure S3 **Effect of GSK3β inhibitor on pancreatic cancer cell migration.** The time course for cell migration was minitored by monolayer-based wound healing assay for MIA PaCa-2 and BxPC-3 cells in the presence of DMSO or AR-A014418 (AR 5 µM, AR 10 µM). The relative widths of wounds were measured and expressed as a percentage of the initial gap at time zero. Values are means ± SD of three separate experiments. *p<0.05, statistically significant difference between cells treated with DMSO or AR-A014418.
(TIF)

Table S1 **Clinical and pathologic characteristics of patients with pancreatic cancer.**
(DOC)

Table S2 **Primary antibodies used for Western blotting and Sequences of the primers used for RT-PCR amplification.**
(DOC)

Table S3 **Comparison of IC_50_ values of gemcitabine and a GSK3β inhibitor (AR-A014418) between pancreatic cancer cell lines (PANC-1, MIA PaCa-2 and BxPC-3).**
(DOC)

Table S4 **Changes in 50% cell survival inhibitory concentration (IC_50_) of gemcitabine in combination with different doses of AR-A014418 in pancreatic cancer.**
(DOC)
