# Supplementary figures and images for: Aberrant Glycogen Synthase Kinase 3β Is Involved in Pancreatic Cancer Cell Invasion and Resistance to Therapy
Source: PLoS One. 2013 Feb 8;8(2):e55289. doi: 10.1371/journal.pone.0055289 (PMC3568118; doi:10.1371/journal.pone.0055289)

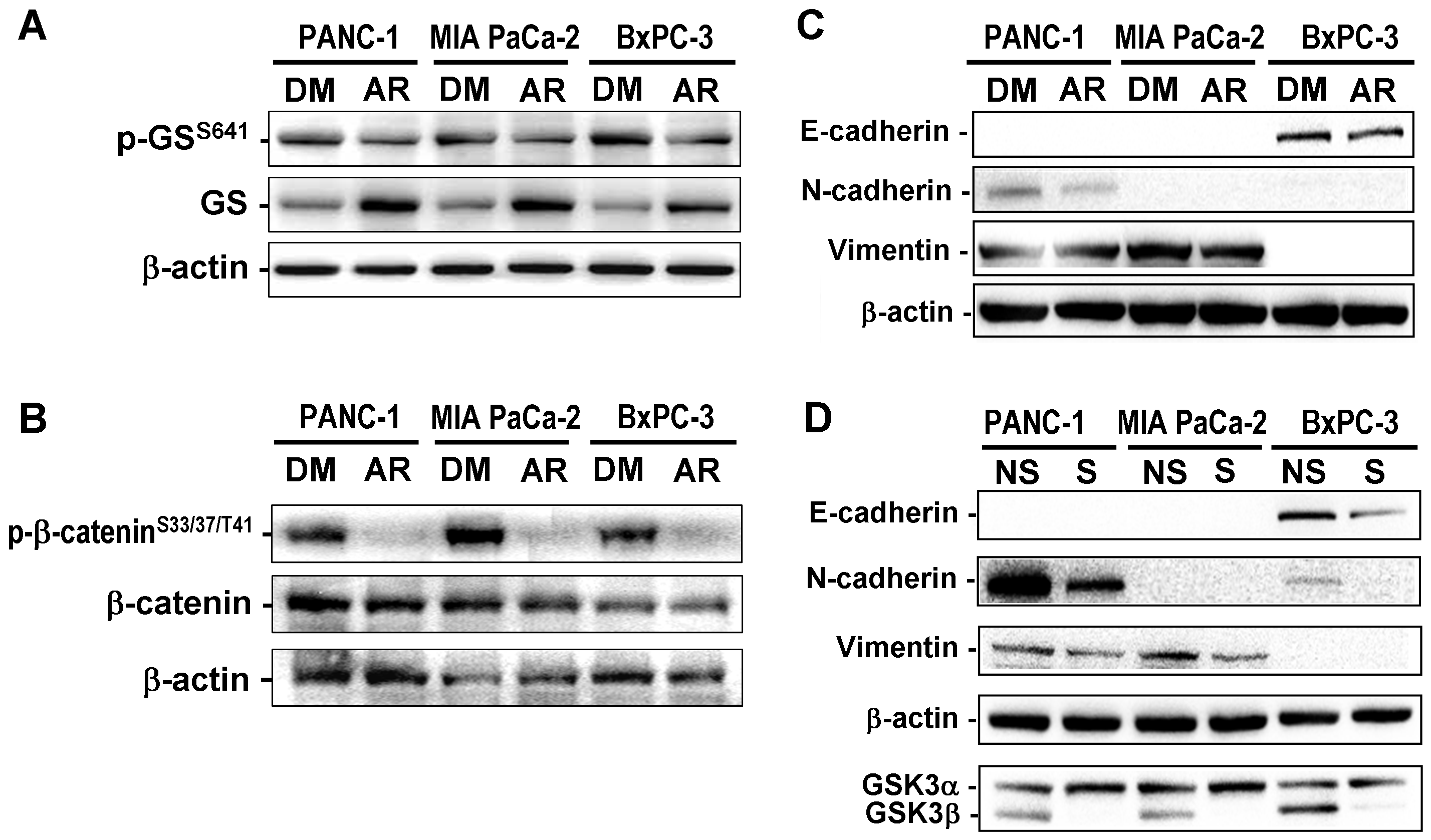

Supplement: Figure S1 — Effects of GSK3β inhibition on expression and phosphorylation of the proteins in pancreatic cancer cells. The levels of expression and phosphorylation of the indicated proteins were examined by Western blotting in pancreatic cancer cells after treatment with the respective agents. (A, B) Expression of GS and β-catenin and their phosphorylation (p-GSS641, p-β-catenin S33/37/T41) were examined and compared between the same pancreatic cancer cells treated with DMSO (DM) or 10 µM AR-A014418 (AR) for 6 hrs. (C) Expression of E-cadherin, N-cadherin and vimentin in pancreatic cancer cells treated with DMSO (DM) or 10 µM AR-A014418 (AR) for 6 hrs. (D) Expression of E-cadherin, N-cadherin, vimentin and GSK3α and GSK3β in pancreatic cancer cells transfected with non-specific siRNA (NS) or GSK3β-specific (S) siRNA (10 nM each). (A–D) The amount of protein extract in each sample was monitored by expression of β-actin. (TIF) [file pone.0055289.s001.tif]

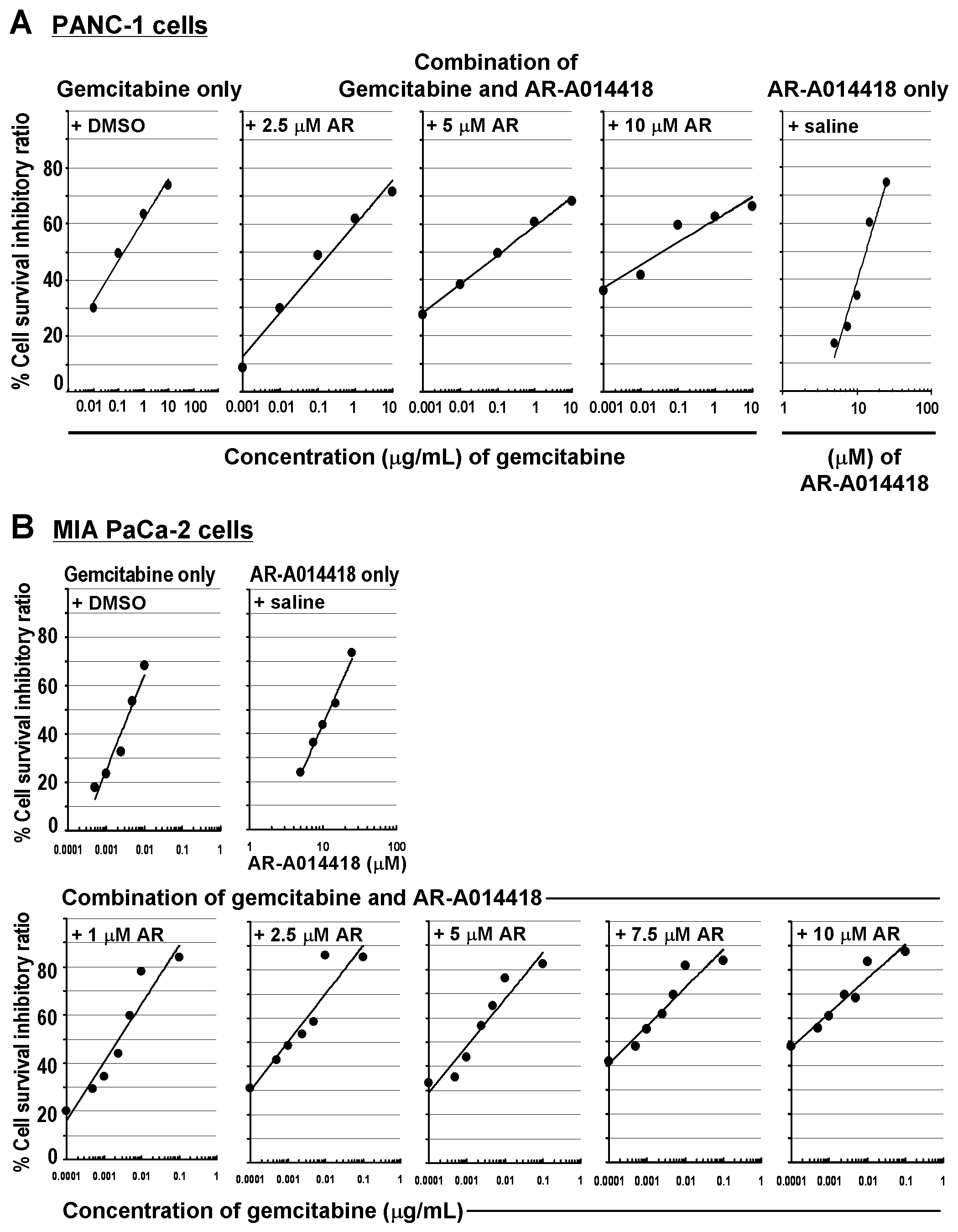

Supplement: Figure S2 — Effects of gemciatbine and AR-A014418, alone or in combination, against pancreatic cancer cells. Inhibitory effects of gemcitabine, AR-A014418 and combinations of the two agents at different doses were examined on the survival of pancreatic cancer cells. PANC-1 (A) and MIA PaCa-2 (B) cells were treated with escalating doses of either gemcitabine, AR-A014418 or both agents in combination at the doses indicated. Relative (%) cell survival ratios for each cell line were examined by WST-8 assay at 48 hrs after treatment with the respective agent. IC50 of gemcitabine in the absence (+ DMSO) or presence of AR-A014418 (+ AR) at the indicated doses was determined and is shown in Table S4. (TIF) [file pone.0055289.s002.tif]

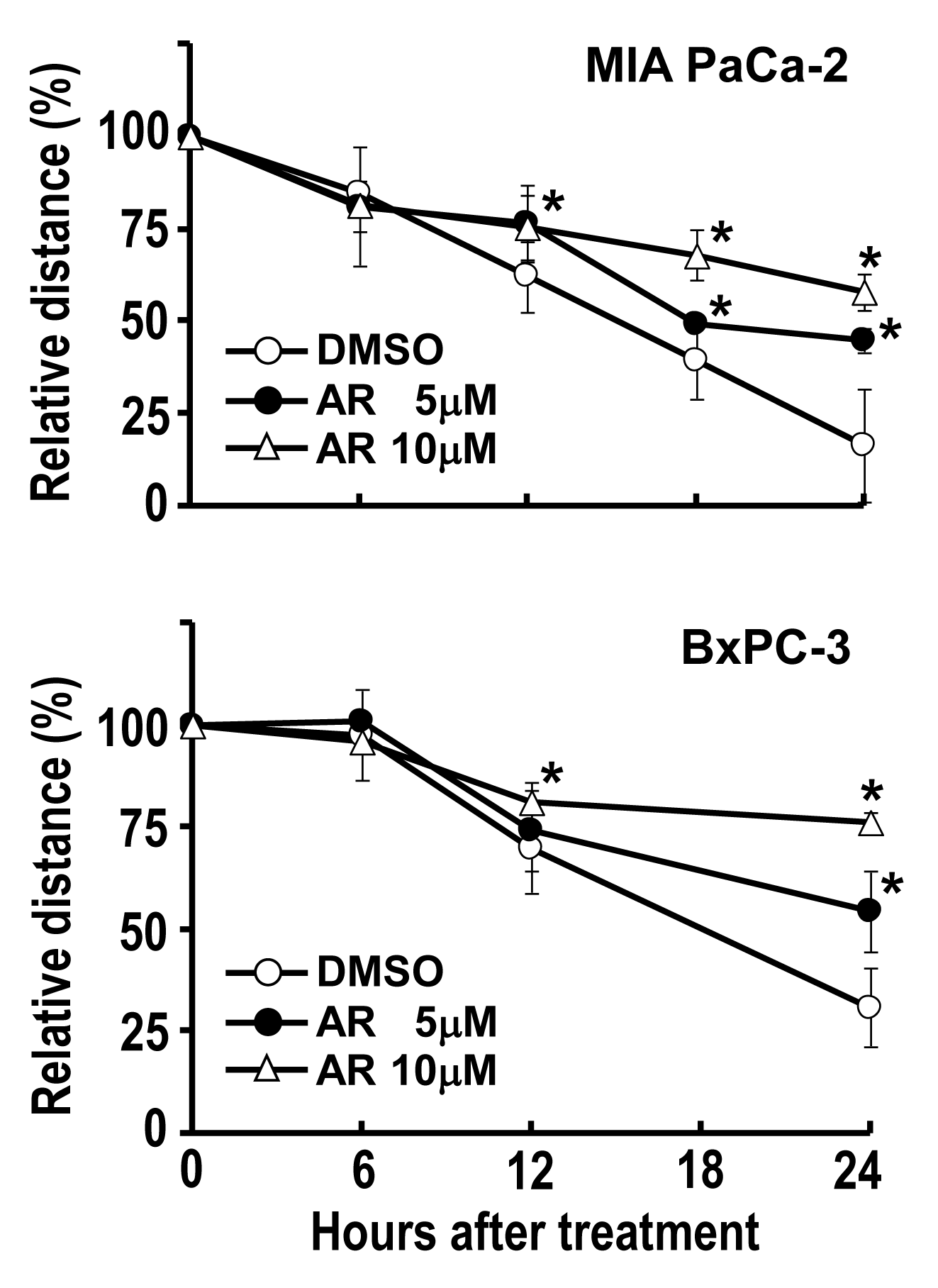

Supplement: Figure S3 — Effect of GSK3β inhibitor on pancreatic cancer cell migration. The time course for cell migration was minitored by monolayer-based wound healing assay for MIA PaCa-2 and BxPC-3 cells in the presence of DMSO or AR-A014418 (AR 5 µM, AR 10 µM). The relative widths of wounds were measured and expressed as a percentage of the initial gap at time zero. Values are means ± SD of three separate experiments. *p<0.05, statistically significant difference between cells treated with DMSO or AR-A014418. (TIF) [file pone.0055289.s003.tif]
