# Supplementary material for: Aberrant Glycogen Synthase Kinase 3β Is Involved in Pancreatic Cancer Cell Invasion and Resistance to Therapy
Source: PLoS One. 2013 Feb 8;8(2):e55289. doi: 10.1371/journal.pone.0055289 (PMC3568118; doi:10.1371/journal.pone.0055289)
Supplement: Table S1 — Clinical and pathologic characteristics of patients with pancreatic cancer. (DOC) [file pone.0055289.s004.doc]

**Supporting Table S1.** Clinical and pathologic characteristics of patients with pancreatic cancer

| Patient No. | Age/Sex | Site of tumor in pancreas | TNM status at surgery | Stage | Treatment | Higher expression / phosphorylation | | | | |
| --- | --- | --- | --- | --- | --- | --- | --- | --- | --- | --- |
|  |  |  |  |  |  | GSK3β | p-GSK3β^Y216^ | MMP-2 | p-FAK^Y397^ | p-FAK^Y861^ |
| 1 | 65/F | head | Tis N0 M0 | 0 | PD | (–) | (–) | (–) | (–) | (–) |
| 2 | 74/F | head | T3 N0 M0 | IIA | PD | (–) | (–) | (+) | (+) | (+) |
| 3 | 72/F | head | T3 N0 M0 | IIA | PD | (+) | (+) | (+) | (+) | (+) |
| 4 | 67/F | head | T2 N0 M0 | IB | PD | (–) | (–) | (–) | (–) | (–) |
| 5 | 71/F | head | T3 N1 M0 | IIB | PD | (+) | (+) | (+) | (+) | (+) |
| 6 | 76/M | head | T4 N1 M0 | III | PD | (+) | (+) | (+) | (+) | (+) |
| 7 | 51/M | head | T2 N0 M0 | IB | PD | (–) | (–) | (+) | (–) | (–) |
| 8 | 60/F | head | T4 N1 M0 | III | Bypass  tumor biopsy | (+) | (+) | (+) | (+) | (+) |
| 9 | 65/M | head | T3 N1 M0 | IIB | PD | (+) | (+) | (+) | (+) | (+) |
| 10 | 70/F | head | T3 N0 M0 | IIA | PD | (–) | (–) | (+) | (–) | (–) |
| 11 | 55/M | head | T4 N1 M1 | IV | Bypass,  tumor biopsy | (+) | (+) | (+) | (+) | (+) |
| 12 | 69/F | body | T1 N0 M0 | IA | DP  splenectomy | (–) | (–) | (–) | (–) | (–) |
| 13 | 76/M | head | T1 N0 M0 | IA | PD | (–) | (–) | (–) | (–) | (–) |
| 14 | 56/M | body | T3 N1 M0 | IIB | DP  splenectomy | (+) | (+) | (+) | (+) | (+) |
| 15 | 56/M | tail | T3 N1 M0 | IIB | DP  splenectomy | (+) | (+) | (+) | (+) | (+) |

Abbreviations: F, female; M, male; PD, pancreatoduodenectomy; DP, distal pancreatectomy
