# Supplementary material for: Aberrant Glycogen Synthase Kinase 3β Is Involved in Pancreatic Cancer Cell Invasion and Resistance to Therapy
Source: PLoS One. 2013 Feb 8;8(2):e55289. doi: 10.1371/journal.pone.0055289 (PMC3568118; doi:10.1371/journal.pone.0055289)
Supplement: Table S2 — Primary antibodies used for Western blotting and Sequences of the primers used for RT-PCR amplification. (DOC) [file pone.0055289.s005.doc]

**Supporting Table S2.** Primary antibodies used for Western blotting and Sequences of the primers used for RT-PCR amplification

| Antibodies to | Working dilutions | Sources (Companies) |
| --- | --- | --- |
| GSK3α/β | 1:1,000 | Upstate Biotechnology |
| GSK3β | 1:1,000  1:100 for IHC | BD Biosciences |
| p-GSK3β^S9^ | 1:1,000 | Cell Signaling Technology |
| p-GSK3β^Y216^ | 1:1,000  1:100 for IHC | Cell Signaling Technology |
| β-catenin | 1:1,000  1:100 for IHC | BD Biosciences |
| p-β-catenin^S33/37/T41^ | 1:1,000  1:100 for IHC | Cell Signaling Technology |
| glycogen synthase | 1:1,000 | Cell Signaling Technology |
| p-GS^S641^ | 1:1,000 | Gene Tex |
| cyclin D1 | 1:1,000 | Cell Signaling Technology |
| CDK4 | 1:1,000 | Cell Signaling Technology |
| CDK6 | 1:1,000 | Cell Signaling Technology |
| Rb | 1:1,000 | Cell Signaling Technology |
| p-Rb^S780^ | 1:1,000 | Cell Signaling Technology |
| p-Rb^S807/811^ | 1:1,000 | Cell Signaling Technology |
| E-cadherin | 1:2,000 | BD Biosciences |
| N-cadherin | 1:2,000 | BD Biosciences |
| vimentin | 1:5,000 | BD Biosciences |
| Rac1 | 1:1,000  1:200 for IFS | Thermo  BD Biosciences |
| FAK | 1:2,000  1:100 for IHC | Cell Signaling Technology |
| p-FAK^Y397^ | 1:2,000  1:100 for IHC | Invitrogen |
| p-FAK^Y861^ | 1:2,000  1:100 for IHC | Invitrogen |
| MMP-2 | 1:200 for IHC | Merch |
| β-actin | 1:4,000 | Ambion |
| Genes | Primers | Sequences |
| MMP-2 | Sense primer | 5’-AGCTCCCGGAAAAGATTGATG-3’ |
|  | Anti-sense primer | 5’-CAGGGTGCTGGCTGAGTAGAT-3’ |
| β-actin | Sense primer | 5’-ATTGCCGACAGGATGCAGA-3’ |
|  | Anti-sense primer | 5’-GAGTACTTGCGCTCAGGAGGA-3’ |

Working dilutions: for Western blotting unless otherwise indicated

IHC: immunohistochemistry; IFS: immunofluorescence staining

Abbreviations: p-β-catenin^S33/37/T41^, β-catenin phosphorylated at serine (S) 33, S37 and/or threonine (T) 41; CDK, cyclin dependent kinase; FAK, focal adhesion kinase; p-FAK^Y397^, FAK phosphorylated at tyrosine (Y) 397; p-FAK^Y861^, FAK phosphorylated at Y861; GS, glycogen synthase; p-GS^S641^, GS phosphorylated at S641; GSK3, glycogen synthase kinase 3; p-GSK3β^S9^, GSK3β phosphorylated at S9; p-GSK3β^Y216^, GSK3β phosphorylated at Y216; MMP-2, matrix metalloproteinase-2; Rb, retinoblastoma gene/protein; p-Rb^S780^, Rb phosphorylated at S780; p-Rb^S807/811^, Rb phosphorylated at S807 and/or S811.
