# Supplementary material for: Aberrant Glycogen Synthase Kinase 3β Is Involved in Pancreatic Cancer Cell Invasion and Resistance to Therapy
Source: PLoS One. 2013 Feb 8;8(2):e55289. doi: 10.1371/journal.pone.0055289 (PMC3568118; doi:10.1371/journal.pone.0055289)
Supplement: Table S3 — Comparison of IC50 values of gemcitabine and a GSK3β inhibitor (AR-A014418) between pancreatic cancer cell lines (PANC-1, MIA PaCa-2 and BxPC-3). (DOC) [file pone.0055289.s006.doc]

**Supporting Table S3.** Comparison of IC_50_ values of gemcitabine and a GSK3β inhibitor (AR-A014418) between pancreatic cancer cell lines (PANC-1, MIA PaCa-2 and BxPC-3)

|  | IC_50_ | |
| --- | --- | --- |
| Cell lines | Gemcitabine (ng/mL) | AR-A014418 (μM) |
| PANC-1 | 163.5 | 13.2 |
| MIA PaCa-2 | 59.3 | 17.0 |
| BxPC-3 | 4.3 | 12.3 |

In each cell line, IC_50_ was calculated by measuring relative cell viability in the presence of PBS or escalating concentrations of gemcitabine for 72 hrs, and in the presence of DMSO or AR-A014418 at escalating concentrations for 72 hrs.
