# Supplementary material for: Aberrant Glycogen Synthase Kinase 3β Is Involved in Pancreatic Cancer Cell Invasion and Resistance to Therapy
Source: PLoS One. 2013 Feb 8;8(2):e55289. doi: 10.1371/journal.pone.0055289 (PMC3568118; doi:10.1371/journal.pone.0055289)
Supplement: Table S4 — Changes in 50% cell survival inhibitory concentration (IC50) of gemcitabine in combination with different doses of AR-A014418 in pancreatic cancer. (DOC) [file pone.0055289.s007.doc]

**Supporting Table S4.** Changes in 50% cell survival inhibitory concentration (IC_50_) of gemcitabine in combination with different doses of AR-A014418 in pancreatic cancer cells.

| Cell line | AR-A014418 (μM) | IC50 of gemcitabine (ng/mL) |
| --- | --- | --- |
| PANC-1 | 0 | 163.4 |
|  | 2.5 | 240.3 |
|  | 5 | 130.2 |
|  | 10 | 39.5 |
| BxPC-3 | 0 | 4.33 |
|  | 1.0 | 2.52 |
|  | 2.5 | 1.02 |
|  | 5 | 1.22 |
|  | 7.5 | 0.38 |
|  | 10 | 0.15 |
